# Acoustic Emission Biomarkers for the Detection and Monitoring of Early Knee Osteoarthritis: Protocol for a Prospective, Single-Center, Exploratory Study

**DOI:** 10.2196/67032

**Published:** 2026-02-26

**Authors:** Louis Leuthard, Jérôme Thevenot, Tomás Teijeiro, David Atienza, Vincent A Stadelmann

**Affiliations:** 1 Department of Research and Development, Schulthess Klinik, Lengghalde 2, Zurich, CH-8008, Switzerland, +41 76 321 6954; 2 Embedded Systems Laboratory, École Polytechnique Fédérale de Lausanne, Lausanne, Switzerland; 3 Basque Center for Applied Mathematics, Bilbao, Spain

**Keywords:** joint health, multimodal, joint sound analysis, prognosis, explorative study, feature extraction, disease progression

## Abstract

**Background:**

Osteoarthritis is a highly prevalent and disabling condition. In early stages, patients are asymptomatic or only experience activity-related pain. When pain intensifies, the disease has often progressed, with few treatment options besides knee arthroplasty. Recently, there has been a growing interest in acoustic emissions generated by poorly lubricated and/or damaged moving joint surfaces, as seen in osteoarthritis. Noninvasive analysis of knee sound could help identify patients with early-stage osteoarthritis at a low cost, without radiation exposure. Thus, preventive measures could be implemented earlier and delay the progression of osteoarthritis.

**Objective:**

This study aims to identify acoustic biomarkers with prognostic value for the early detection of osteoarthritis. To achieve this, a preliminary reference database will be developed using a subpopulation at risk of developing osteoarthritis, incorporating acoustic signals and gold-standard clinical measures for validation.

**Methods:**

A total of 100 patients with previous reconstructive knee surgery and 20 healthy controls will be examined twice at a 9-month interval for both knees. Acoustic emissions (AE) will be recorded with the Inmodi knee brace (École Polytechnique Fédérale de Lausanne; EPFL) during 4 functional tests (unloaded flexion-extension, sit-to-stand test, one-step test, and walk test). Gold-standard osteoarthritis diagnostics will be assessed and evaluated by an experienced radiologist for (1) the magnetic resonance imaging (MRI) Osteoarthritis Knee Score (MOAKS) and (2) the Kellgren-Lawrence (KL) grading scores. Feature analysis and multivariate modeling will be used to study the association between the extracted sound parameters and osteoarthritis (MOAKS and KL). In addition, thermal images will be taken to investigate the state of inflammation. Participants will fill out 5 health-related questionnaires (Oxford Knee Score [OKS], Core Outcome Measures Index [COMI], University of California at Los Angeles [UCLA ]activity scale, Knee Injury and Osteoarthritis Outcome Score [KOOS], and Forgotten Joint Knee Score-12 [FJS-12]) at both time points.

**Results:**

Recruitment and data collection started in December 2022. In November 2023, data collection of all 120 participants (female: n=49, 41%) was completed for the first visit. The data collection for the second visit will end in 2024. Preliminary data processing and analysis are ongoing at the time of this writing.

**Conclusions:**

This exploratory study will contribute to a better understanding of the disease progression of osteoarthritis and the use of acoustic biomarkers for predicting osteoarthritis.

## Introduction

### Disease Background

Osteoarthritis is a highly prevalent and disabling condition that affects over 7% of people globally (528 million people) [1]. It is significantly limiting their mobility and independent lifestyle. Osteoarthritis is mainly characterized by loss of cartilage, structural changes in bone, and inflammation of the synovium and joint capsule. Common risk factors include aging, obesity, prior joint injury, and repetitive joint stress increasing local joint wear. Osteoarthritis takes several years to develop before the patient sees the doctor when the pain intensifies. In the early stages, patients are asymptomatic or only experience activity-related pain. The pain becomes constant over time with intermittent intense pain episodes. The average annual cost of osteoarthritis for an individual is estimated to be between US $700-$15,600 worldwide (2019) [1]. Consequently, osteoarthritis has a large socioeconomic impact due to its associated high medical cost expenditures, as well as indirect costs, such as early retirement and high absenteeism from work.

Osteoarthritis typically affects the hips, knees, hands, feet, and spine, with a high prevalence of polyarticular involvement. This study focuses on knee osteoarthritis.

### Contemporary Standard of Assessment

The contemporary gold-standard assessment of the severity of knee osteoarthritis is based on plain radiography, using the Kellgren-Lawrence (KL) grading system, which was accepted as a standard by the World Health Organization in 1961 [2]. This system does not evaluate the primary affected tissue—cartilage—but only the overall aspect of the joint. It is, therefore, unable to detect early stages of osteoarthritis, and the diagnosis is often established at a late stage, limiting the number of treatment options and typically leading to knee replacement surgery.

### Alternative Assessment Methods

Earlier diagnosis and improved patient stratification are required to deploy preventive osteoarthritis treatments. Possible early diagnostic options include magnetic resonance imaging (MRI) and biochemical markers. MRI can be used to assess changes in cartilage volume, thickness, or even quality [3]. However, the use of MRI is limited by its high cost, availability, and the absence of a validated international score [4]. Alternative clinical imaging modalities have also been proposed to address the shortcomings of KL at the time of writing this, such as ultrasound [5] or computed tomography [6]; however, their use remains relatively limited compared to X-rays. Many biochemical markers are under investigation, but so far, they lack specificity to joint tissues and to particular joints, and sensitivity and specificity are still not high enough for widespread clinical use [7]. Recently, there has been a growing interest in acoustic analysis as an alternative to conventional biomarkers, particularly for the knee joint. The assumption is that the affected joint will generate abnormal acoustic events during motion due to poor lubrication or surface damage and that acoustic analysis will detect and quantify these abnormal events as a measure of joint damage [8-17].

### Relevance of the Project

A wearable device was developed at the École Polytechnique Fédérale de Lausanne (EPFL) for collecting and processing acoustic and kinematic signals. This device—called the Inmodi knee brace—collects airborne acoustic data from the medial and lateral subpatellar areas and kinematic information from the thigh and the calf, both modalities synchronously [17,18]. The platform is then complemented by thermal information of the knee as a measure of inflammation and uses artificial intelligence–based data analysis to extract and combine relevant biomarkers of joint function from each modality. Preliminary data suggests that this combined approach may enable a noninvasive early diagnosis of osteoarthritis. A preliminary *in vitro* animal study with horses’ legs showed a strong association between the joint condition and the power of acoustic emission (AE) signals analyzed [19]. Recently, we tested a prototype of the Inmodi knee brace in 17 patients from our clinic shortly before total knee arthroplasty (TKA). These patients endured severe osteoarthritis on the leg undergoing surgery and mild to moderate on the contralateral one. A machine learning (ML) algorithm was able to discriminate the leg states with a specificity of 0.96 (perfect=1; data not yet published).

The aim of this pilot project is to further explore the potential of the Inmodi system to diagnose osteoarthritis at earlier stages. Therefore, we will develop a preliminary reference database in a subpopulation at risk of developing osteoarthritis, while following recent recommendations [20]. The identification and validation of novel biomarkers with a ML algorithm require a training dataset and a test dataset, both with potential biomarkers and gold-standard clinical outcomes for validation. This database will require a sample size large enough to be statistically sound to prevent statistical overfitting. Unfortunately, power analysis is not possible for most ML methods, due to the impossibility of guaranteeing statistical assumptions on the distributions of the features and their linear and nonlinear dependencies. That is why we rely on leave-one-subject out nested cross-validation for assessing the statistical relevance of the resulting models. This is the most conservative and robust cross-validation strategy for overfitting. The specific sample size of 120 was considered as feasible within the time constraints of the project.

Noninvasive knee health diagnostics should help identify patients at risk at a relatively low cost and without radiation exposure and enable preventive measures to be implemented earlier than with standard radiographic imaging.

## Methods

### Project Design

This is a prospective, single-center, exploratory study. A cross-sectional and a longitudinal dataset will be collected. A total of 120 participants will be recruited and examined twice at a 9-month interval at the Schulthess Klinik in Zurich (see Table 1 for the measurement procedure). The cross-sectional dataset stems from the first visit of the participants and serves for the identification of AE biomarkers for osteoarthritis. The longitudinal dataset combines the data from the first and second visits and tests whether the osteoarthritis changes can be assessed from the biomarkers. A secondary aim is to evaluate the ability of the AE biomarkers from the first visit to predict the condition’s progression as observed during the second visit.

**Table 1.** Summary table with all project visits, including relevant procedures and timelines.

Study period	Information	Screening	1st visit	2nd visit
Time point	Precall (maximum 2 months)	0	0-2 months	9 months (±2 months)
Oral and written information	✓			
Written consent		✓		
Check inclusion/ exclusion criteria		✓		
Anthropometric and patient data			✓	✓
EOS radiography			✓	✓
MRI^a^			✓	✓
Acoustic emission test with Inmodi knee brace			✓	✓
PROMs^b^ (at home)			✓	✓

a MRI: magnetic resonance imaging.

b PROM: patient reported-outcome measurement.

### Project Population

This study targets a population at risk of osteoarthritis. The risk of knee osteoarthritis after knee joint injury is up to 5-fold that of noninjured patients. As many as 50% of individuals with an anterior cruciate ligament (ACL) or meniscus tear develop knee osteoarthritis [21]. Therefore, to represent the whole distribution of osteoarthritis stages in the study sample, participants will be recruited from the clinic’s Meniscus/ACL/Cartilage (MAC) surgery registry at 2- and 5-year postoperative times. All patients from the MAC registry will be screened regardless of whether they have undergone isolated meniscal tear repair, isolated ligament reconstruction, or a combined meniscal and ligament procedure. Additionally, a healthy control group and a late-stage osteoarthritis group will also be included. The inclusion and exclusion criteria presented in Textbox 1, follow established standards and are consistent with those used in similar studies (eg, NCT02937064).

The total study sample will be 120 patients in 6 groups:

- Group A: 20 healthy participants
- Group B1: 20 patients from the MAC registry at 2 years postoperative with normal outcomes (normal and poor outcomes are defined in section Recruitment)
- Group B2: 20 patients from the MAC registry at 2 years postoperative with poor outcomes
- Group C1: 20 patients from the MAC registry at 5 years postoperative with normal outcomes
- Group C2: 20 patients from the MAC registry at 5 years postoperative with poor outcomes
- Group D: 20 patients booked for in-house unilateral TKA surgery for severe osteoarthritis

Even if it is not possible to perform an exact power analysis for the ML models to be trained, we can use a simple proxy model to estimate the effect size that we can reasonably expect to detect with a sample size of 120. Thus, if we aim to estimate the differences in KL gradings from the AEs, the smallest detectable correlation with 80% power and a significance level of *P*=.05 would be 0.25. In one-way ANOVA, this corresponds to a minimum explained variance of approximately 7%, which is considered a small-to-medium effect size [22]. Smaller effect sizes are unlikely to be detected within our population.

Textbox 1. **Detailed inclusion and exclusion criteria for the groups.**

**Inclusion criteria**

Group A:

- Aged 35-65 years
- People judge themselves to be of good subjective health without any knee problems

Groups B and C:

- Aged 35-65 years
- Unilateral knee surgery (other side as internal control)
- Participants in Meniscus/ACL/Cartilage (MAC) surgery registry
- Completed patient-reported outcome measurements (PROM) questionnaires at 2 years or 5 years within the last 6 months

Group D:

- Aged 35-75 years
- Patient booked for total knee arthroplasty (TKA) due to severe osteoarthritis

**Exclusion criteria**

- Inability to give consent or follow procedures
- No understanding of the German language
- Open wounds or tissue injuries
- Irritated or infected sections on the limbs
- Class II obesity is defined by BMI ≥35 kg/m2 (comorbidities associated with obesity should be investigated in future studies)
- Uncooperative patients who disregard or cannot follow instructions, including those who abuse drugs and/or alcohol
- Pregnant or with the intention to get pregnant (X-rays)
- Current address outside of Switzerland
- Groups B and C only: revision surgeries at the operated knee, death, known pathologies, or former injuries of the comparator knee

### Recruitment

A subject will be considered enrolled in the study only after providing written consent. No study-specific data will be collected until then. The recruitment procedures for the sample groups are described below:

- For Group A, healthy controls will be recruited via the standard outlets, such as the clinic’s website and social media accounts.
- For groups B and C, patients’ medical files from the MAC registry of our clinic will be screened for inclusion and exclusion criteria. Based on the known distributions of PROM scores (instrument: Core Outcome Measures Index [COMI] [23]) in the registry at 2 and 5 years, the patients’ scores will be categorized as normal/poor scores.

- For groups B1 and C1, lists of patients will be generated and ordered by age and sex. Beginning with each patient nearest to age 35, 38, 41, 44, 47, 50, 53, 56, 59, and 62 years, the following recruitment procedure will occur: if no exclusion reason was found, the patient will be contacted by phone by the study nurse and offered study participation. If the patient refuses or cannot be reached after 3 attempts of contact, the next patient within the same age group will be screened and contacted. This ensures a balanced age distribution within sex within the groups. Lists of patients for groups B2 and C2 (by sex) will be generated and ordered by COMI score (decreasing), and patients will be contacted successively.
- The poor outcomes group B2 consists of patients with scores ≥2.
- The normal outcomes group B1 is defined as patients closely around the median COMI scores of their respective group (determined medians are: 2-year, females, 0.9; 2-year, males, 0.7; 5-year, females, 0.5; 5-year, males, 0.5).

- For Group D, all patients scheduled for unilateral TKA surgery at the site and fulfilling the inclusion criteria will be informed about the study and asked for participation. Screening is done by the study manager as soon as the operation is booked. If the patient is eligible, the study nurse will inform the patient by phone about the study and ask for participation. If the patient is interested, a brochure with information about the study will be sent in written form to the patient to consider participation carefully at home for at least 24 hours. This will be done successively until the sample size of 10 males and 10 females is reached.

### Study Procedures

The overall study duration is 2 years. Recruitment will last for 6 months. For a patient, the duration of the study is 9 months, with one visit at baseline and one visit at 9 months. All measurements will occur at the clinic, including anthropometric data, clinical examination, and functional and temperature assessments. Additionally, the participants should fill in PROMs, which are provided as an online questionnaire via the Research Electronic Data Capture (REDCap; Vanderbilt University) application. If preferred, a paper version of the questionnaire can be provided to the patient. Participants can do this at home.

### Anthropometric and Patient Data

The following data will be collected from the patient’s medical file: sex, age, height, weight, BMI, body fat percentage, comorbidities, and adverse events since the knee operation.

#### EOS Radiography

Long-leg radiographic images will be acquired in a standing position as stereoradiography using the EOS Edge (EOS X-Ray Imaging Acquisition System). The photon counting detector allows for a dose reduction by a factor between 8 and 10 compared to conventional X-ray radiography [24,25]. EOS imaging of the knee has been validated for osteoarthritis grading using KL grading scores [26].

For all participants, bilateral long-leg, standing EOS images in anteroposterior view will be retrieved and analyzed for:

- Osteoarthritis grading (KL grade 0‐4)
- Compartmental joint space width
- Leg alignment angles:
  - Knee joint alignment (normal, varus, and valgus) measured with hip-knee-ankle angle
  - Mechanical axis length
  - Proximal tibia width
  - Mechanical axis deviation
  - Joint angles
  - Mechanical lateral distal femoral angle
  - Mechanical medial proximal tibial angle

#### MRI Technique

Both knees of the participants will be scanned using a 3 Tesla MRI scanner (Magnetom Prisma; Siemens) without any contrast agent (Table 2). MRI allows visualization and assessment of structural changes in joint tissues, such as cartilage, meniscus, and subchondral bone. A radiologist will use the MRI Osteoarthritis Knee Score (MOAKS) system to score these degenerative structures in osteoarthritis semiquantitatively. The MOAKS score has demonstrated very good to excellent reliability [27] and grades tissues in 7 different subregions of the knee [28].

**Table 2.** Acquisition parameters of magnetic resonance imaging (MRI) sequences.

MRI^a^ sequence	STIR^b^	T1 TSE^c^	PD^d^ dixon	PD TSE FS^e^
Orientation	coronal	coronal	3D [4,5]	3D [6,7]
Field of view (mm)	154	160	160	160
Repetition time (ms)	4850	558	5010	4200
Echo time (ms)	31	7.1	44	42
Number of slices	28	28	34	31
Slice thickness (mm)	3	3	2.5	2.7
Distance factor (%)	20	15	15	10
Voxel area (mm^3^)	0.6×0.6×3	0.5×0.5×3	0.4×0.4×2.5	0.4×0.4×2.7
Pixel bandwidth (Hz/Px)	260	340	334	260
Flip angles (deg°)	180	180	180	180

a MRI: magnetic resonance imaging.

b STIR: short tau inversion recovery.

c TSE: turbo spin echo.

d PD: proton density.

e FS: fat saturation.

#### AE Test With Inmodi Knee Brace

**Figure 1.**
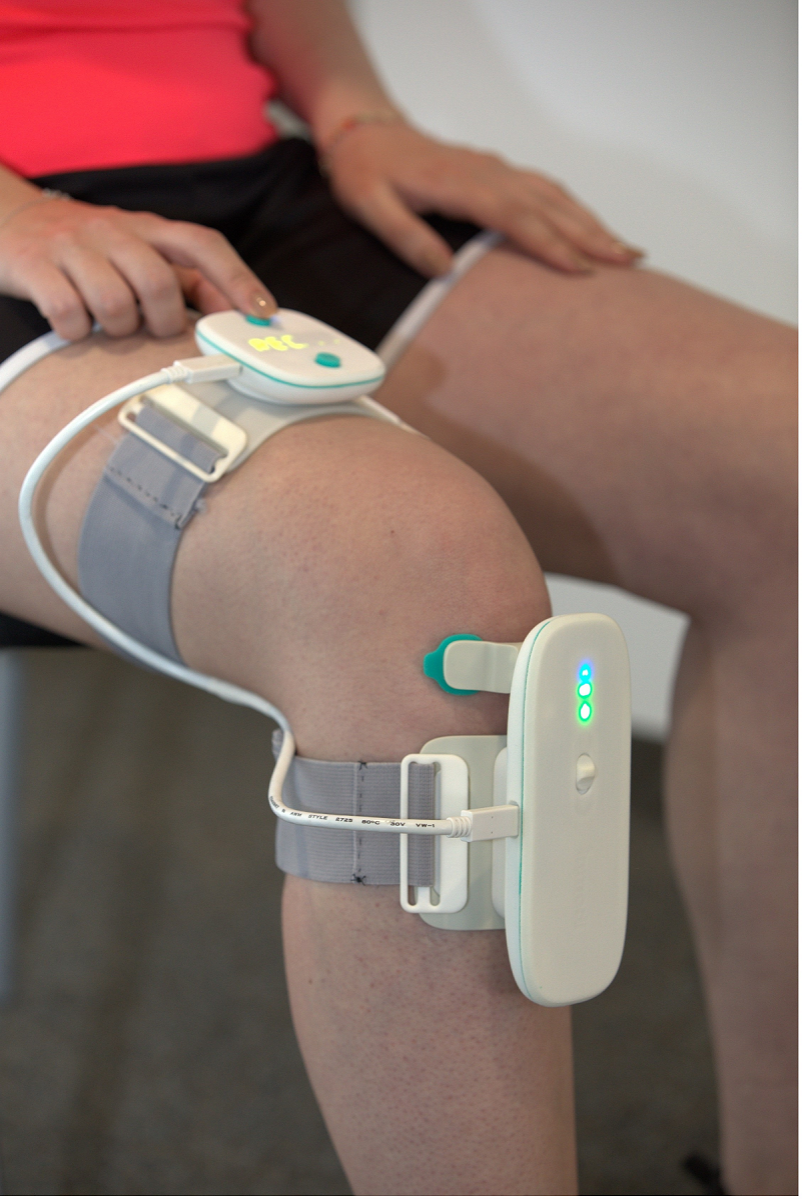
Upper part (positioned on the thigh) and lower part (positioned on the calf) of the Inmodi knee brace.

**Figure 2.**
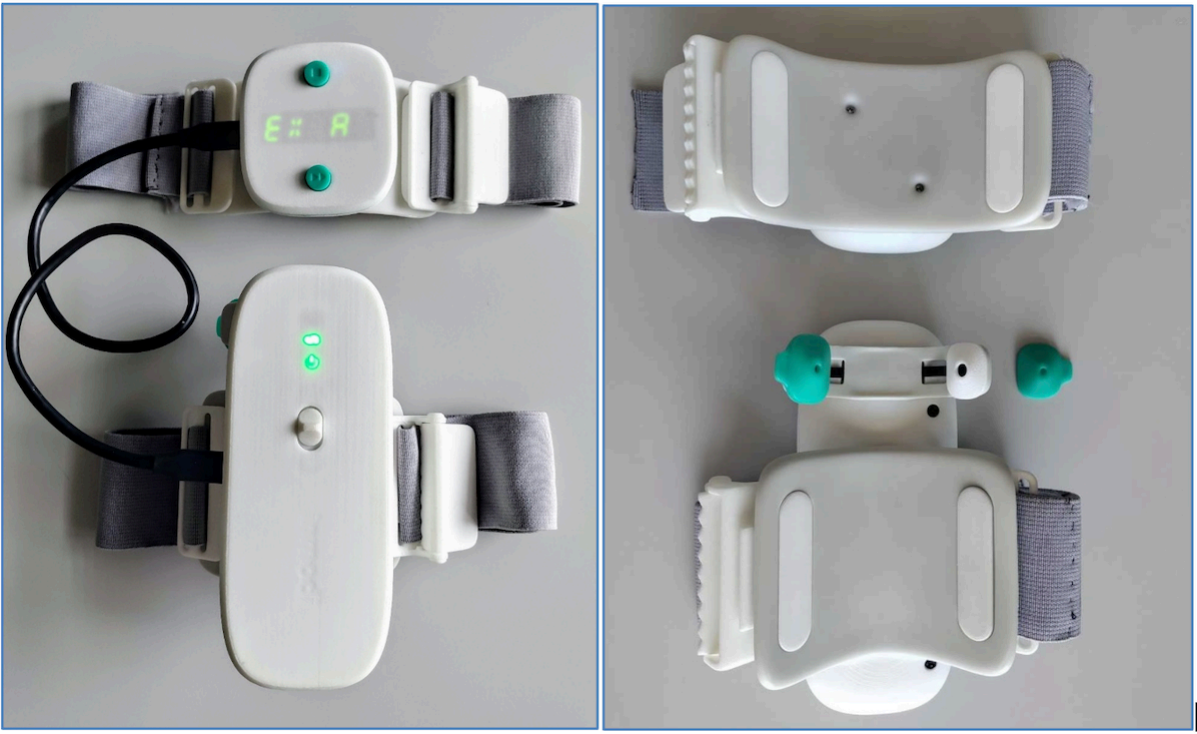
The Inmodi device from the top (left) and the bottom (right). The green silicone covers can be removed from the microphone frames for cleaning.

The tests will be done with the Inmodi knee brace (v2; Figure 1) for AE assessment of both knees simultaneously [18]. The brace is designed to not hinder movement and is made of biocompatible materials (polylactic acid/thermoplastic polyurethane) covered by silicon commonly used in orthopedics for direct skin contact. All the embedded electronics are fully isolated, without possible contact with the patient. The device consists of 2 parts to be attached on the limb with the help of straps (Figure 2):

- The main part is to be positioned on the calf. This part has an embedded inertial measurement unit (IMU) module to collect kinematic data on the lower part of the limb and 2 microphones positioned in an enclosure covered by removable silicone. The microphones are placed at each extremity of a flexible arch to be positioned on the medial and lateral subpatellar regions. This mainframe also has a battery, microcontroller, and embedded flash memory to save the raw data.
- A secondary part to be positioned on the thighs and connected by USB to the main part. This secondary part has an IMU module to collect the kinematics of the upper part of the limb and an interface with buttons to start/stop the recording of all sensors.

All the sensors collect data in a synchronized manner: the microphones (SPH0645LM4H-B; Knowles) have a sampling rate of 22000 Hz, and the IMU (BNO080; CEVA Hillcrest Labs) has a frequency of 100 Hz. The microphones were chosen to have a sampling rate covering the relevant acoustic information suggested in the literature [9,11,15]. We decided to use airborne microphones to improve the device’s usability for easy positioning without needing extra tapes to maintain contact sensors on the skin and potential associated rubbing artifacts from them. The literature has reported that, despite having a lower signal-to-noise ratio, airborne AEs can be collected by Micro-electro-mechanical systems microphones as an alternative to contact sensors to assess knee health [15]. The advantage of using air microphones over contact microphones resides in the clinical applicability of the solution, which requires minimal preparation from the medical workers. However, such an approach requires a design to ensure a consistent distance between the airborne microphone and the skin to keep the microphone’s sensitivity to collect knee sounds. The Inmodi device was designed to keep the microphone at a distance of 6.5 mm from the skin while preventing any skin contact with the microphone membrane, thanks to a rigid frame encapsulating the microphone covered by a silicone piece. The silicone piece is the only part in contact with the skin, providing a damping of artifact noise due to possible friction, and it is removable for cleaning between patients.

The tests are based on recommendations by the Osteoarthritis Research Society International (OARSI) for people with knee osteoarthritis [29]:

- Unloaded flexion-extension (F-E) of the knee in a seated position, starting with a knee angle of 90°, followed by a full extension and returning to the starting position (6 times). A video will guide the pace of the motion to normalize the velocity among patients (comfortable speed).
- Sit-to-stand test (STS) at a comfortable pace (6 times). Force plates (FP-BTA; Vernier) will be used during this exercise to assess the ground reaction force for each sole and detect any asymmetrical loading of the joints.
- One-step test (OST) is an exercise step platform, which will be used to perform a complete ascent and descent at a comfortable speed (6 times per leg).
- Walk test at a comfortable pace (10m ×2 times).

The following measurements will be performed together with the AE tests:

- Temperature assessment: thermal distribution of the knee joint will be assessed with a far infrared thermal sensor array (MLX90640; Melexis) to evaluate the state of inflammation. Localized thermal images will be recorded at three time points: (1) at the beginning of the whole test, just after arriving, (2) after warming up (5 minutes on a stationary bicycle), and (3) at the end of all tests.
- Pain assessment: after the test session, the level of knee pain will be assessed using a visual analog scale. Participants will quantify the maximum knee pain experienced during the tests for each knee separately by placing a vertical mark on a 10 cm horizontal line. The line ranges from 0 (“no pain at all”) to 10 (“not endurable pain”).

#### PROMs

The following PROMs questionnaires were sent out by email or paper form depending on participants’ preference and were completed in their homes.

- Oxford Knee Score (OKS): this instrument contains 12 questions about an individual’s activities of daily living and how they have been affected by pain over the last 4 weeks. Its summed score ranges from 0 (severe functional problems) to 48 (excellent knee function) [30,31].
- Core Outcome Measures Index (COMI) knee: assesses the main outcomes of importance to patients with knee problems (pain, function, symptom-specific well-being, quality of life, disability). It is a 6-item multidimensional instrument with 5 domain scores for pain (item 1), function (item 2), symptom-specific well-being (item 3), general quality of life (item 4), and disability (average of items 5 and 6) are averaged to give a COMI score ranging from 0 to 10 (the higher the score, the worse the status) [32].
- University of California at Los Angeles (UCLA): an activity rating scale, which rates the activity level of joint replacement patients globally from 1 to 10. The patient indicates her/his most appropriate activity level (level 1 = “no physical activity, dependent on others”; level 10 = “regular participation in impact sports”) [33].
- Knee Injury and Osteoarthritis Outcome Score (KOOS): an instrument to assess the patient’s opinion about their knee and associated problems. There are 5 subscales with individual sum scores normalized to 0-100 (0 = “extreme symptoms”; 100 = “no symptoms”): pain, other symptoms, function in daily living, function in sport and recreation, and knee-related quality of life. A total score of all subscales combined has not been validated [34].
- Forgotten Joint Score 12 (FJS-12) is a concise 12-item PROM that evaluates patients’ ability to forget their joints daily. Each item is answered within a 5-point Likert scale with the following response options: never (0); almost never (1); seldom (2); sometimes (3); and mostly (4) [35].

### Signal Segmentation and Denoising

This involves the isolation of the different movement phases of the acoustic signals, the removal of frequency components of low interest, and the separation of the acoustic signal into different sources (sounds from the knee, ambient sounds, sounds from the device, etc). The segmentation will be based mostly on the kinematic data, using the relative angles between the upper and lower parts of the device to set the beginning and end time of each repetitive movement. For denoising, most previous literature suggests relevant acoustic information is below 20kHz, and typically a bandpass filtering from 100 Hz up to 10‐16,ooo Hz is applied depending on the study [9,11,15]. We will start from this approach, relying on standard digital filters such as Butterworth filters and complementary transforms such as wavelets. Different cutoff frequencies (3, 6, 8, 10, and 16,000 Hz) will be explored to minimize the amount of data to process and simplify feature extraction. Data separation will be achieved through techniques such as empirical mode decomposition and independent component analysis. To reinforce this separation, a further clustering analysis will be performed with techniques such as Density-based spatial clustering of applications with noise to try to unveil robust causal relations between audio segments and sources.

### Feature Extraction

This stage involves the calculation of the outcome parameters that will allow the distinction of different osteoarthritis grades. We will initially rely on state-of-the-art parameters in audio classification, such as spectral analysis (mel-frequency cepstrum coefficients [MFCC]) [36,37] and fractality (the First log-cumulant of the scaling exponent, Global Holder Exponent; paper submitted), average level, peak magnitude (peak amplitude) [38] and click patterns (number of clicks/hits), and number of clicks passing threshold [39].

We will also explore the development of novel features specifically oriented to osteoarthritis classification, such as matched filters of prototype sounds [40].

### Feature Analysis and Multivariate Modeling

In this stage we will study the association between the extracted parameters and osteoarthritis (using the KL or MOAKS grading as the dependent variable), both individually for each feature and also by creating linear and nonlinear multivariate models. For this, we will follow a strategy guided by the principles of (1) increasing complexity and (2) clinical explainability. Thus, the initial models we consider are linear discriminant analysis and logistic regression, which would be ideal options in case we have a reduced number of highly informative and discriminative features. In this ideal scenario, the models would be highly interpretable and easily translatable to a clinical description. In case more sophisticated models are required, we prioritize ensembles of decision trees, such as random forest and gradient boosting, due to the possibility of performing advanced feature importance analysis and derivation of statistical decision rules.

All features will be tested for normality by Shapiro-Wilk tests and presented as mean (SD; normally distributed data) or median and range and, if needed, in histograms, box plots, or violin plots (nonnormal data).

Associations between AE parameters and osteoarthritis (KL or MOAKS grading) will be evaluated using Pearson (normally distributed data) or Spearman (nonnormal data) repeated measures correlation coefficients, as well as with logistic regression models. Mixed ANOVA will be used to compare differences in the AE, osteoarthritis grading parameters between groups. Also, we will specifically assess the relevance of potential confounding variables such as BMI, age, activity level, and gender. The significance level will be set to *P*=.05, and due to the potentially large number of tests, we will apply the Holm post-hoc correction to all *P* values. For multivariate models, the statistical association will be characterized using leave-one-subject-out nested cross-validation, with a subsequent estimation of a confidence interval for prediction accuracy. This is the most conservative and robust cross-validation strategy for overfitting , and with our sample size (n=120), we will have a good estimation of the model variance. Statistical analyses will be done with R (version 4; R Foundation), Matlab (version 2021b or higher; MathWorks, Inc), and Python (version 3.6 or higher; Python Software Foundation).

### Statistics

Since this is an exploratory study, the statistical data analysis will also be mostly exploratory, so multiple techniques and models will be assessed and compared. Overall, the analysis of acoustic signals includes signal segmentation and denoising, feature extraction, feature analysis, and multivariate modeling.

To ensure the reproducibility of the experimental results, we will provide open-source access to the exact feature sets and analysis code for the reported results.

### Withdrawal and Discontinuation

The patients can withdraw from the study at any point in time. In the declaration of consent, the patient agrees that the data already collected stays in the study. Patients who have knee surgery or a knee injury within the follow-up time or patients who become otherwise severely ill will not be invited for the second visit. In all cases above, the data will be encrypted and used for evaluation. If the enrollment phase is still ongoing, the withdrawn patient will be replaced with a person of the same sex, comparable age, and study group type (A, B1, B2, C1, C2, and D).

If a participant misses the first visit as well as a substitute appointment, the participant will be withdrawn and replaced. If a participant misses the second visit and substitute appointment, his data collected from the first visit will be used.

If a person becomes pregnant, the study will continue but without EOS radiography.

### Handling of Missing Data

Patients who fail to appear for the MRI, EOS, or AE test with the Inmodi knee brace will be invited again. After the second miss, they will be excluded (and replaced if the recruitment phase is still ongoing). Missing source data from MRI or EOS are not expected. In the case of missing readouts in the electronic case report form, the radiologist is asked to do the missing readout or to give reasons for the missing.

In case of unexpected data loss or data damage with the Inmodi brace data collection, the participant will be reinvited.

Concerning PROMs, a maximum of 2 missing items is allowed in OKS. They will be imputed with the mean of all other responses. In case of additional missing items, the patient will be contacted to clarify. For COMI and UCLA, missing data will not be imputed. The patient will be contacted for clarification. For KOOS: If more than 50% of subscale items are missing, the subscale score cannot be calculated. Otherwise, the sum of the items answered is normalized to 0‐100. For FJS-12, if more than 4 of the answers are missing, the total score cannot be calculated. With fewer items missing, these can be replaced by the mean of the items answered. Generally, if a patient makes more than one entry for a question, the worst applies.

### Ethical Considerations

An ethical permit was granted for this study by the Business Administration System for Ethical Committees (BASEC No.: 2022‐01699) from the ethics committee of Canton Zurich, and written informed consent was obtained from all subjects after the nature and possible consequences of the studies were explained. The participants did not receive any compensation. All study data were stored in a password-protected REDCap database where participants are only identified by a unique identifier. A key list is kept secure by the project leader. An integrated audit trail system maintains a record of initial entries and changes (reason for change, date and time of change, and user identification). All procedures performed in this study were developed internally to suit our specific clinical context and have been validated through prior successful implementation [41] and are in line with the 1964 Helsinki Declaration and its amendments. This study was registered at Clinicaltrials.gov with study identifier NCT06351059.

## Results

Recruitment and data collection started in December 2022. In November 2023, data collection has been completed for the first visit. The data collection for the second visit will end in 2024. Data processing and analysis are ongoing at the time of this writing.

Baseline data of study participants are shown in Table 3. The median age for the healthy controls (A) is slightly lower, and for the TKA group (D) slightly higher compared to the 2- and 5-year postoperative patients (B and C). Older patients in the TKA group (D) are reasonable, as TKA surgery is often the last option left for treatment. We do observe higher values for BMI in the poor postoperative outcome groups (B2 and C2) and the TKA group (D). Balanced sex distribution was not achieved in 2- and 5-year postoperative groups due to the low willingness to participate as well as the low numbers of female patients within our registry meeting all the inclusion criteria.

**Table 3.** Baseline data of study participants.

Characteristic	A (n=20)	B1 (n=20)	B2 (n=20)	C1 (n=20)	C2 (n=20)	D (n=20)
Age, mean (SD)	46 (7)	50 (8)	54 (8)	52 (7)	52 (7)	67 (7)
BMI, mean (SD)	24.25 (2.82)	24.75 (2.75)	26.05 (2.44)	24.65 (2.31)	26.00 (3.71)	26.35 (3.99)
Sex, n (%)						
Female	10 (50)	9 (45)	8 (40)	6 (30)	6 (30)	10 (50)
Male	10 (50)	11 (55)	12 (60)	14 (70)	14 (70)	10 (50)

## Discussion

### Principal Findings

This exploratory study aims to identify biomarkers with a prognostic value in the prediction of osteoarthritis. Acoustic, kinematic, and thermal features will be extracted and combined with an artificial intelligence algorithm, and evaluated against gold-standard osteoarthritis diagnosis, such as radiographs and MRI. Some candidate acoustic biomarkers have been identified in a preliminary study [9], using a multifractal analysis method. However, multiple other candidates will be evaluated individually or in combination in our study. Therefore, the primary endpoint can be defined as the area under the curve of the receiver operating characteristic analysis of each biomarker or combination of biomarkers in the regression analysis, finally identifying the parameters with the highest sensitivity and specificity to predict early-stage osteoarthritis in clinical use. AE is patient-dependent, and as such, demographic and baseline health data will be captured and entered as covariates in the regression models. The secondary objective is to evaluate the value of these biomarkers in predicting osteoarthritis evolution. For this objective, the patients will be examined a second time at 9-month intervals, and the new biomarkers will be introduced in regression models to evaluate the predicted performance for osteoarthritis evolution.

### Limitations

One major limitation of this study is the sample size and distribution. Although many patients participate in our institutional MAC registry, the inclusion criteria narrow down the eligible patients. Furthermore, the willingness of participants was lower than expected, which influenced the distribution of age and sex within the different groups. Whether these variations in baseline characteristics do influence the identification of acoustic biomarkers with a prognostic value for early-stage osteoarthritis, remains unclear until the data analysis has been completed. Still, the sample size is significantly larger than most comparable studies [20] and in line with the most relevant references in the state-of-the-art [16].

A second limitation of the study is the recruitment of patients postsurgery reconstruction. We decided to choose this patient population, as they have a higher predisposition to develop osteoarthritis; however, it might limit the generalization of the approach to highly active individuals who are more prone to injury. Furthermore, while having a balanced age and BMI between groups reduces the impact of covariates, it also limits the generalization of the approach to populations with different osteoarthritis phenotypes. To further enhance the approach’s potential, this pilot study should be applied to other centers for data collection to expand the sample size for validation with other osteoarthritis populations with different backgrounds.

Another concern is that resulting acoustic biomarkers may mostly reflect movement patterns rather than a causal relation to osteoarthritis. This critical aspect has been carefully considered in the design of the experimental protocol. As described in the study procedures section, the exercises (movements) the participants must perform are extremely standardized. Also, we will have the kinematic data associated with any potentially discriminative AEs. This allows us to study any movement patterns that may be associated with acoustic biomarkers and assess how strongly they are correlated with specific movements that may not be specific for osteoarthritis. Also, on top of the IMU in the device, sit-to-stand is performed on force plates, so an imbalanced load could also be accounted for in the models. On the other hand, we believe that finding movement patterns that are strongly correlated with the osteoarthritis gradings would be a very relevant contribution of the study, even if causal links cannot be directly established.

Finally, another limitation is the lack of medical history. Although patients are screened and asked about revision or previous surgeries as well as former injuries of the comparator knee, there is no guarantee a patient has not already been operated on or operated on again at a different hospital.

### Conclusion

The reference database that will be gathered by this study in a subpopulation at risk of developing osteoarthritis will help to further explore the potential of acoustic biomarkers to diagnose osteoarthritis at earlier stages. A noninvasive knee sound diagnostic tool could provide a better understanding of the disease and its progression at low cost and without radiation exposure. Therefore, this study will contribute to improved clinical decision-making and enable preventive measures.

## Supplementary material

10.2196/67032 Checklist 1 SPIRIT (Standard Protocol Items: Recommendations for Interventional Trials) checklist.

## Abbreviations

ACL: anterior cruciate ligament
AE: acoustic emission
BASEC: Business Administration System for Ethical Committees
COMI: Core Outcome Measures Index
EPFL: École Polytechnique Fédérale de Lausanne
FJS-12: Forgotten Joint Score (with 12 items)
IMU: inertial measurement module
KL: Kellgren-Lawrence
KOOS: Knee Injury and Osteoarthritis Outcome Score
MAC: Meniscus/ACL/Cartilage-surgery registry
ML: machine learning
MOAKS: MRI Osteoarthritis Knee Score
MRI: magnetic resonance imaging
OARSI: Osteoarthritis Research Society International
OKS: Oxford Knee Score
PROM: patient reported outcome measurement
REDCap: Research Electronic Data Capture
TKA: total knee arthroplasty
UCLA: University of California at Los Angeles
